# Increased expression of T cell immunoglobulin and mucin domain 3 aggravates brain inflammation via regulation of the function of microglia/macrophages after intracerebral hemorrhage in mice

**DOI:** 10.1186/1742-2094-10-141

**Published:** 2013-12-01

**Authors:** ChangJun Xu, Tao Wang, Si Cheng, YuGuang Liu

**Affiliations:** 1 Department of Neurosurgery, Qilu Hospital of Shandong University, No.107 Wenhuaxi road, Jinan, Shandong 250012, PR China

**Keywords:** T cell immunoglobulin, Mucin domain 3, Brain inflammation, Microglia, Macrophages, TNF-α, IL-1β

## Abstract

**Background:**

Microglia/macrophages are known to play important roles in initiating brain inflammation after spontaneous intracerebral hemorrhage (ICH). T cell immunoglobulin and mucin domain-3 (Tim-3) have been proven to play a critical part in several inflammatory diseases through regulation of both adaptive and innate immune responses. Tim-3 can be expressed by microglia/macrophages and regulates their function in the innate immune response. However, the effect of Tim-3 on inflammatory responses following ICH is unclear.

**Methods:**

In this study, we investigated Tim-3 expression, the inflammatory cytokines tumor necrosis factor-α (TNF-α) and interleukin-1β (IL-1β), and brain water content in peri-hematomal brain tissue at 12 hours and at 1, 3, 5, and 7 days post-ICH in wild type (WT) ICH and Tim-3^−/−^ ICH mice. The numbers of Tim-3 positive cells,astrocytes, neutrophils and microglia/macrophages were detected using immunofluorescence staining. Cytokines were measured by ELISA. Double immunoflurorescence labeling was performed to identify the cellular source of Tim-3 expression. Mouse neurological deficit scores were assessed through animal behavior.

**Results:**

Expression of Tim-3 increased early in mouse peri-hematomal brain tissue after autologous blood injection, peaked at day 1, and was positively correlated with the concentrations of TNF-α, IL-1β, and brain water content. Tim-3 was predominantly expressed in microglia/macrophages. Compared with WT mice, Tim-3^−/−^ mice had reduced ICH-induced brain inflammation with decreased TNF-α and IL-1β, cerebral edema and neurological deficit scores. Moreover, Tim-/- inhibited activation of microglia/macrophages. The number of activated microglia/macrophages in Tim-3^−/−^ ICH mice was much lower than that in WT ICH mice.

**Conclusions:**

Our findings demonstrate that Tim-3 plays an important role in brain inflammation after ICH, and may be a potential treatment target.

## Background

Spontaneous intracerebral hemorrhage (ICH) is a common disease with high mortality and morbidity, accounting for 15 to 20% of all stokes and affecting more than 2 million people worldwide each year [1,2]. The mortality rate of ICH is more than 40% and only 20% of survivors can live independently within six months [3]. But until now, there has been no satisfactory treatment in clinical practice, mainly because the mechanisms of brain damage after ICH are unclear. Increasing researches show that inflammatory response plays an important role in ICH-induced secondary brain damage [4,5]. Brain inflammation after ICH is characterized by accumulation of activated inflammatory cells, such as blood-derived cells (macrophages, leukocytes) and brain resident cells (astrocytes, microglia and mast cells). These reactive cells can release inflammatory mediators, including chemokines, cytokine, protease, prostaglandins and other immunoactive molecules [6,7]. The microglia are the first cells to react to brain damage among all the inflammatory cells. Activated microglia have a similar shape to the blood-derived macrophages, therefore microglia are also called as the brain macrophages. Increasing evidence indicates that microglia/macrophages are activated early following ICH, and release a series of toxic factors, including chemokines, cytokines, reactive oxygen species (ROS), cyclooxygenase-2, protease, heme oxygenase-1 and prostaglandins [8-10]. Therefore, microglia/macrophages play important roles in the secondary brain damage. Tim-3 is a new immuno-regulation molecule found in 2002, which is expressed specially in activated Th1 cells [11,12]. Moreover, it has been proven that Tim-3 is also expressed in the cells of the innate immune system, including macrophages, mast cells and dendritic cells [13,14]. Tim-3 expression in microglia/macrophages can be upregulated and induce the production of proinflammatory cytokines, such as tumor necrosis factor-a (TNF-a) and interleukin-1β (IL-1β), which can aggravate inflammation and secondary brain damage [14]. However, until now, the effect of Tim-3 on inflammatory response following ICH has been unclear. Considering that the microglia/macrophages are the key cells for inducing brain inflammation and secondary brain damage, and that Tim-3 can regulate the function of microglia/macrophages, we hypothesized that Tim-3 possibly took part in ICH-induced inflammation by regulating the function of microglia/macrophages. This experiment was done to prove our hypothesis.

## Materials and methods

### Intracerebral hemorrhage model

The ICH model of mice was established using the method described previously [15]. Animals were anesthetized with chloral hydrate (40 mg/kg, intraperitoneal injection). A catheter plugged in the right femoral artery was used to monitor continuous blood pressure and to take blood sampling. Mice were fixed in a stereotactic frame (Stoelting, Kiel, WI, USA). The puncture point was located 1 mm anterior to the bregma and 2.5 mm lateral to midline. A 1-mm cranial hole was drilled with dental bit and a 27-gauge needle was inserted stereotaxically into the right basal ganglia (4 mm deep). Then, 25 μL of autologous blood was injected using a micro-infusion pump at a rate of 2.5 μL/min. After the infusion, the needle was kept in the place for another 10 minutes to prevent blood leakage, and then the needle was pulled out. The cranial hole was sealed with bone wax and the skin was sutured. Control mice were injected with 25 μL 0.9% saline. Then mice were allowed to recover. During operation, rectal temperature of mice was maintained at 37 ± 1°C. Mean arterial blood pressure (MABP) (mmHg), arterial pH, arterial PO_2_, PCO_2_, Hb (g/L), and glucose levels (mg/dL) were monitored and maintained for stability.

### Animals and grouping

C57BL/6 mice (male, 8 to 10 weeks old, and weight 20 to 25 g) were purchased from the Animal House Center, Medical College of Shangdong University (Jinan, China). Transgenic line Tim-3^−/−^ mice (8 to 10 weeks old, weight 20 to 24 g) were purchased from American Jackson Laboratories (Bar Harbor, ME, USA) and were backcrossed to C57BL/6 mice more than eight times. All animals were housed in individual cages under a 12 hr/12 hr light–dark cycle with temperature 21 ± 1°C, humidity 50 to 60% and free access to food and water. All animals care and experimental protocols were approved by theanimal Ethics Committee of the Sandong University. All efforts were made to minimize the animals’ pain and to reduce the number of animals. Different experimental groups were included: 1) For the WT sham group (n = 60), 25 μL of 0.9% saline was injected into the brain of these mice. 2) For the WT ICH group (n = 60), 25 μL of autologous blood was injected into the right basal ganglia. 3) For the Tim-3^−/−^ sham group (n = 50),the operation was the same as for the WT sham group. 4) For the Tim-3^−/−^ ICH group (n = 50),the operation was the same as for the WT ICH group. A total of 8 mice (5 mice in the WT ICH group and 3 mice in the Tim-3^−/−^ ICH group) died in our experiment, and the number of mice supplemented was the same. There were 228 mice in our experiment.

### Tissue preparation

In the WT sham group and the WT ICH group, 12 mice were anesthetized at each of the following time points: 12 hours and 1, 3, 5, and 7 days after injection. Half of the mice (n = 6) were directly decapitated and their brains were obtained to store at −80°C for use inreal-time RT-PCR, in ELISA and to determine brain water content. The others (n = 6) were used for immunofluorescence. These mice underwent transcardial perfusion with 200 ml of phosphate buffered saline (PBS), followed by 100 ml of 4% paraformaldehyde (PFA) in 0.1 M PBS as described before [16]. The brains were removed and postfixed for 24 hours in 4% PFA, and then were placed in 30% sucrose until sinking. Coronal brain sections of 10 μm thickness were obtained with a freezing microtome (Leica, Nussloch, Germany) and were kept at −20°C. In the Tim-3^−/−^ sham group and Tim-3^−/−^ ICH group, 10 mice were killed at each of the following time points: 12 hours and 1, 3, 5, and 7 days after operation, Among this total, 6 mice were used for real-time RT-PCR, for ELISA and to determine brain water content, and the others (n = 4) were used for immunofluorescence.

### Immunofluorescence staining and cell counting

After being washed in PBS for 10 min, the sections were incubated with 5% bovine serum albumin for 60 minutes in order to block the nonspecific binding, and then were incubated with goat anti-rat Tim-3 primary antibody (1:100; R&D systems, Minneapolis, MN, USA. http://www.rndsystems.com/) at 4°C all the night. After being washed three times with PBS, the sections were incubated with secondary antibody goat anti-rabbit IgG (1:100; KPL, Maryland, USA. http://www.kpl.com/home.cfm) for 60 minutes at the room’s temperature. The sections were rinsed 3 × 5 min and were coverslipped with ProLong antifade medium (Molecular Probes, Eugene, OR, USA). The Tim-3 positive cells were visualized using a fluorescent microscope (Olympus BX51, Japan). For each animal, six representative sections of each brain were selected. Tim-3 positive cells were counted blindly in the approximately 40,000 μm ^2^ of brain tissues around blood clot. In order to further identify the cellular resource of Tim-3 after ICH, double immunofluorescence labeling [9] was performed by simultaneous incubation of goat anti-rat Tim-3 primary antibody with rat anti-mouse CD11b (1:200, eBioscience San Diego, CA, USA. http://www.ebioscience.com/) as marker of activated microglia/macrophage, or rabbit anti-GFAP (1:200, Invitrogen, Carlsbad, CA, USA. http://www.invitrogen.com) as marker of astrocyte, or rabbit anti-MPO (1:100, Dako, Denmark. http://www.dako.com) as mark of neutrophils. In each group, the number of CD11b positive cells (activated microglia/macrophage), GFAP positive cells (astrocyte) and MPO positive cells (neutrophils) were counted using the same method as used for Tim-3 positive cells. Ipp6.0 image processing software was utilized to count the number of Tim-3 positive cells.

### Real-time reverse transcription polymerase chain reaction

According to the methods described previously [15], frozen mice brains were homogenized, and total RNA was obtained from about 4 × 4 × 4 mm^3^ volume of peri-hematomal tissues (blood clot as center under a stereomicroscope) at 12 hours and at 1, 3, 5 and 7 days post-ICH using Trizol reagent (Invitrogen, Carlsbad, CA, USA) in compliance with the manufacture’s instruction. The character of RNA was tested by a spectrophotometer (DU800, Beckman, Palo Alto, CA, USA). The M-MLV Reverse Transcriptase System (Promega, Madison, WI, USA) was performed for reverse transcription. The cDNA was stored at −20°C. Quantitative real-time PCR was fulfilled with a LightCycler (Roche Diagnostics, Mannheim, GM) and with SYBR Green I in SYBR RT-PCR Kit (TaKaRa Biotechnology, Dalian, China) so as to enlarge and detect the expression of Tim-3 mRNA. The transcript amount of the rat β-actin housekeeping gene was quantified as an internal RNA control. Primers were purchased from BioAsia Corp. (Shanghai, China). The primer sequences were as follows: β-actin forward: 5’-GGCATCGTGATGGACTCCG −3’ and β-actin reverse: 5′-GCTGGAAGGTGGACAGCGA −3′; Tim-3 forward: 5′ACTGGTGACCCTCCATAATAACA −3′ and Tim-3 reverse: 5′ATTTTCCTCAGAGCGAATCCT −3′. Experiments were carried out in triplicate for each data point. A threshold cycle value (CT) was calculated by the ΔΔCT method as previously described [17]. The data were analyzed by using Light Cycler Software 4.0 (Roche Diagnostics).

### Enzyme-linked immunosorbent assay

IL-1β and TNF-aare two important inflammatory mediators and can represent the severity of brain inflammation after ICH. The concentrations of IL-1β and TNF-a in brain tissues of the peri-hematomal region were detected via the ELISA method according to the manufacturer’s instructions (R&D systems, Minneapolis, MN, USA). Brain tissues were centrifuged at 12000 rps for 20 min and the supernatant was collected for analysis. The detection threshold of this assay was <1 pg/mL.

### Brain water content

Brain water content indicates the degree of brain edema, which is mainly due to peri-hematomal inflammation and blood–brain barrier breakdown. Brain samples were immediately weighed on an electric analytical balance to get the wet weight, and then dried at 100°C for 24 hours to obtain the dry weight. Brain water content (%) = (wet weight - dry weight)/wet weight of brain tissue × 100.

### Testing of neurological deficit scores

At 12 hours and at 1, 3, 5 and 7 days after operation, the neurofunctional abnormality of mice was tested and scored in each group. An observer blinded to the identity of the mice evaluated behavior. Three behavioral examinations, including ipsilateral circling, forelimb flexion and beam balance were used as described before [18]. Each point is graded from 0 to 4. The maximum abnormal score is 12.

### Statistical analysis

All statistical analyses were performed with SPSS 16.0 for Windows (Chicago, IL, USA). Data are presented as mean ± standard deviation (SD). Multiple group differences were analyzed using one-way or two-way analysis of variance and Student-Newman-Keuls test in post hoc tests. An independent-samples t-test was adopted for comparison of the two groups. The correlation analysis was completed by bivariate. *P <*0.05 was considered as the indication of statistical significance.

## Results

### The physiological parameters in four groups during production of hematoma

During production of hematoma, we monitored rectal temperature, MABP, arterial pH, arterial PO_2_, PCO_2_, Hb, and glucose levels in the four groups. The results showed there was no difference among all groups (*P* > 0.05)(Table 1).

**Table 1:** **Comparison of physiological parameters**^**a**^ (**PP**) **in all groups during production of hematoma**

	**WT sham group (n = 60)**	**WT ICH group (n = 60)**	**Tim-3**^**−/−**^**sham group (n = 50)**	**Tim-3**^**−/−**^**ICH group (n = 50)**
Rectal temperature	36.97 ± 0.54	37.12 ± 0.42	37.06 ± 0.49	37.01 ± 0.39
MABP(mmHg)	100.04 ± 1.86	101.22 ± 1.96	99.75 ± 2.09	100.09 ± 1.66
Arterial pH	7.38 ± 0.04	7.41 ± 0.02	7.40 ± 0.05	7.35 ± 0.02
PO_2_(mmHg)	106.57 ± 8.50	110.21 ± 5.84	109.86 ± 9.71	103.34 ± 5.04
PCO_2_(mmHg)	42.54 ± 4.12	41.58 ± 2.71	41.77 ± 3.07	42.37 ± 3.42
Hb(g/L)	149.17 ± 6.79	147.01 ± 6.86	143.49 ± 8.64	149.06 ± 7.48
Glucose (mg/dL)	135.90 ± 14.76	137.16 ± 8.47	134.64 ± 16.38	137.72 ± 10.08

^a^Data were presented as mean ± standard deviation. The physiological parameters were not differentamong all groups. *P* > 0.05.ICH, intracerebral hemorrhage; Tim-3, T cell immunoglobulin and mucin domain-3; WT, wild type.

### Increase of Tim-3 expression in the peri-hematomal brain tissues

**Figure 1:**
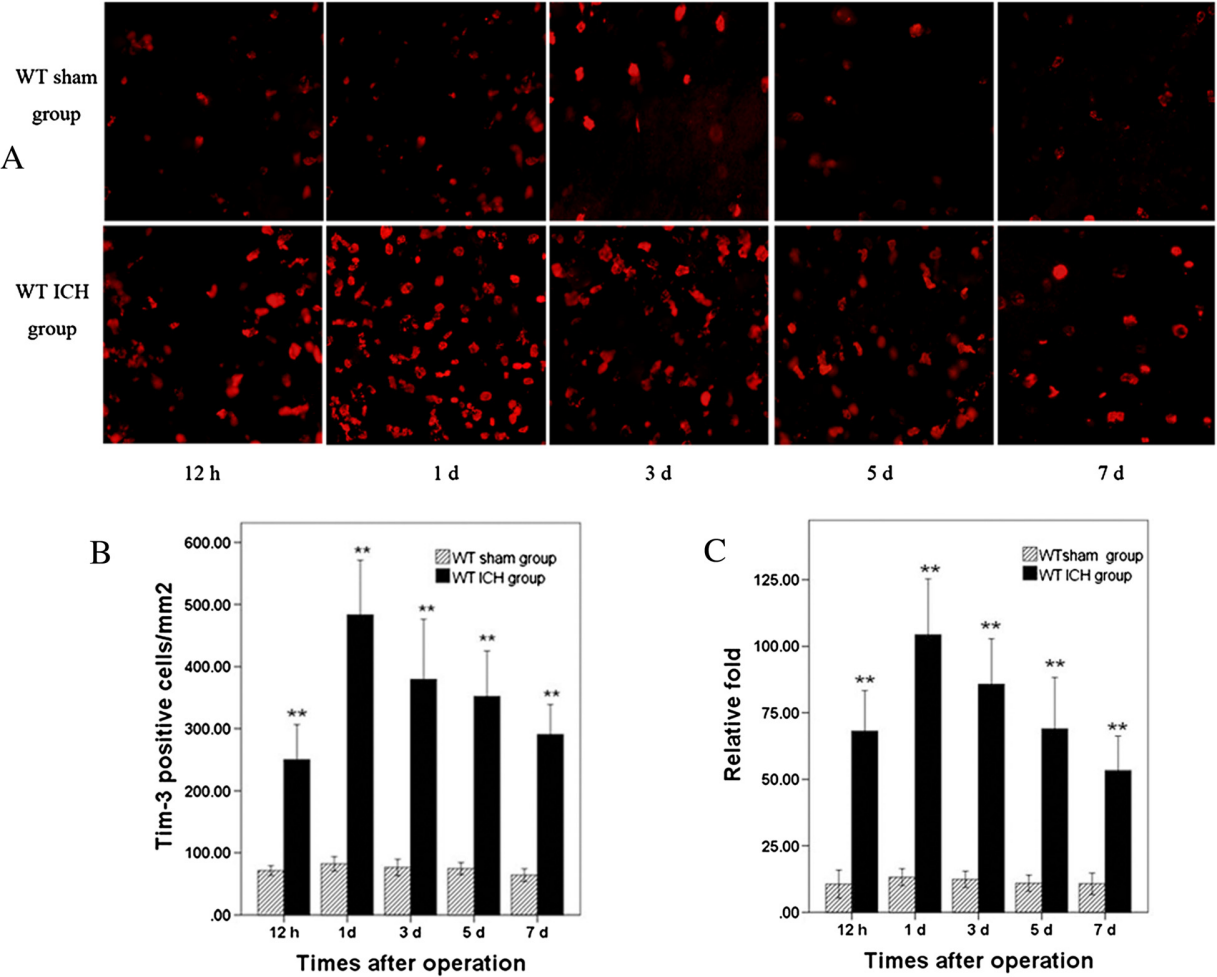
**Expression of Tim-3 positive cells and Tim**-**3 mRNA in peri**-**hematomal brain tissues at time**-**tested points following intracerebral hemorrhage (ICH). A)** and **B)** Immunofluorescence staining showed that the number of Tim-3 positive cells increased at 12 hours in the wild type (WT) ICH group mice (n = 6), with the maximum at Day 1, **P <0.01 versus the WT sham group (mean ± SD). Scale bar = 20 μm. **C)** Real-time RT-PCR indicated that the expression of Tim-3 mRNA increased at all time-tested points in the ICH group mice (n = 6) and peaked at Day 1. **P <0.01 versus WT sham group (mean ± SD). ‘Relative fold’ meant the level of mRNA of Tim-3 relative to that of beta-actin.

In order to investigate the expression of Tim-3 in the peri-hematomal brain tissues, we observed the number of Tim-3 positive cells in the peri-hematomal brain tissues at 12 hours and at 1, 3, 5 and 7 days post-ICH. Results indicated that the number of Tim-3 positive cells in the WT ICH group began to increase at 12 hours, peaked at Day 1, and decreased at Day 3 after ICH. There was a significant difference when compared with that of WT sham mice in all time-tested points (*P* < 0.01) (Figure 1A,B). Furthermore, we studied Tim-3 mRNA expression in the peri-hematomal brain tissues at 12 hours and at 1, 3, 5 and 7 days post-ICH using a real-time RT-PCR method. The change trend of Tim-3 mRNA was similar to the number of Tim-3 positive cells. The Tim-3 mRNA expression was strikingly upregulated in the peri-hematomal brain tissues after ICH at 12 hours, peaked at Day 1, and descended at Day 3. The Tim-3 mRNA expression was significantly different when compared with that in WT sham group(*P* < 0.01) (Figure 1C).

### Preponderant expression of Tim-3 in microglia/macrophages

**Figure 2:**
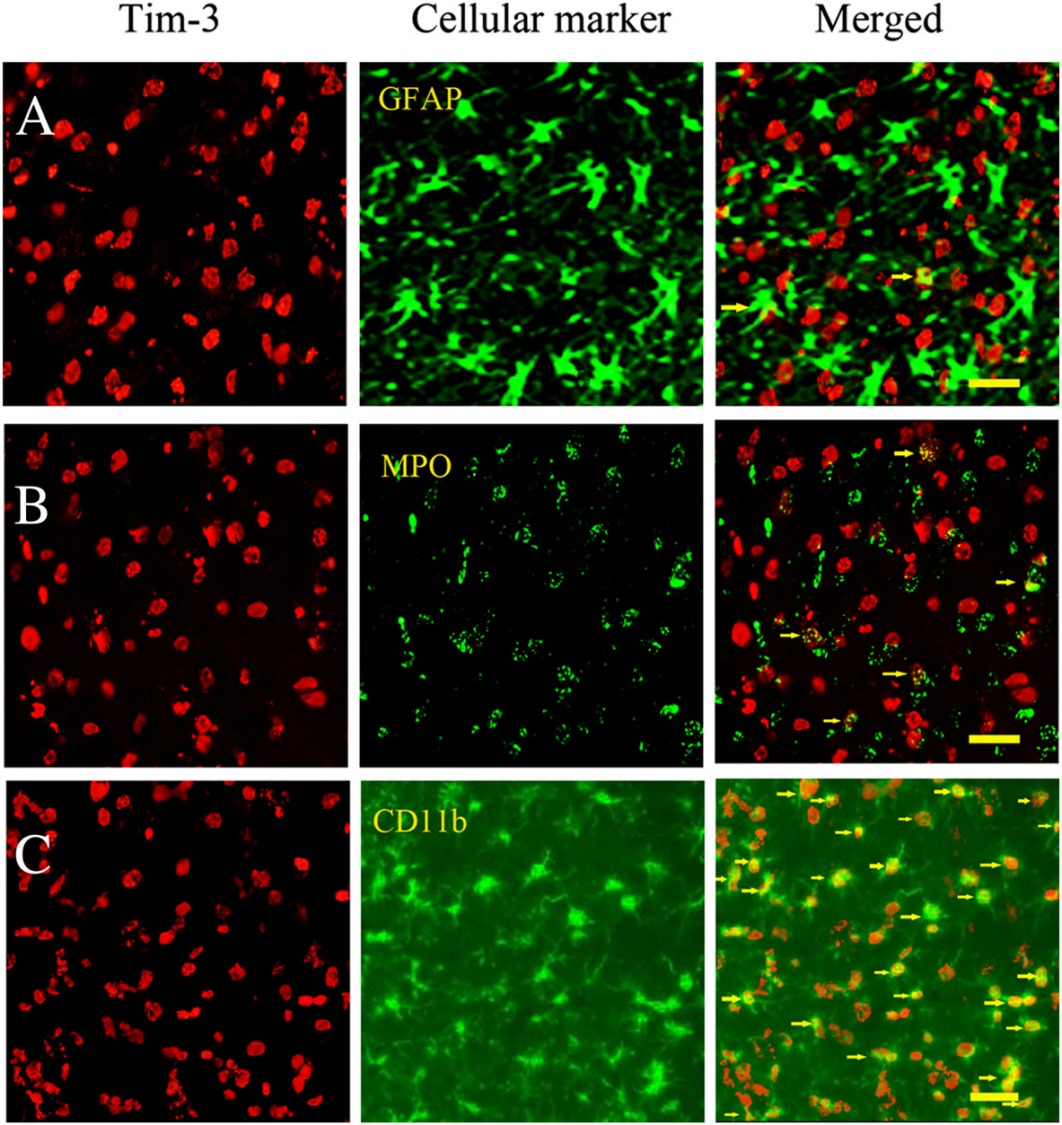
**The expression of Tim**-**3 in different brain cells at day 1 after intracerebral hemorrhage (ICH).** Double-immunofluorescence staining displayed that: **A)** A few of Tim-3 positive cells could express in GFAP^+^ cells (astrocytes). **B)** A few of Tim-3 positive cells could express in MPO^+^ cells (neutrophils), but more than were expressed in GFAP^+^ cells (astrocytes). The arrows indicate co-expressed cells of Tim-3 and MPO. **C)** Almost all CD11b^+^ cells (microglia/macrophages) were Tim-3 positive cells at day 1 after ICH. The arrows indicate co-expressed cells of Tim-3 and CD11b. Scale bar = 20 μm.

To identify the cellular resource of Tim-3 expression, we observed double-immunofluorescence staining. The results revealed that expression of Tim-3 was preponderant in CD11b^+^ cells (microglia/macrophages), was lower in MPO^+^ cells (neutrophils), and was lowest in GFAP^+^ cells (astrocytes) (Figure 2).

### Obvious increase of IL-1β, TNF-a and brain water content in the peri-hematomal brain tissues

**Figure 3:**
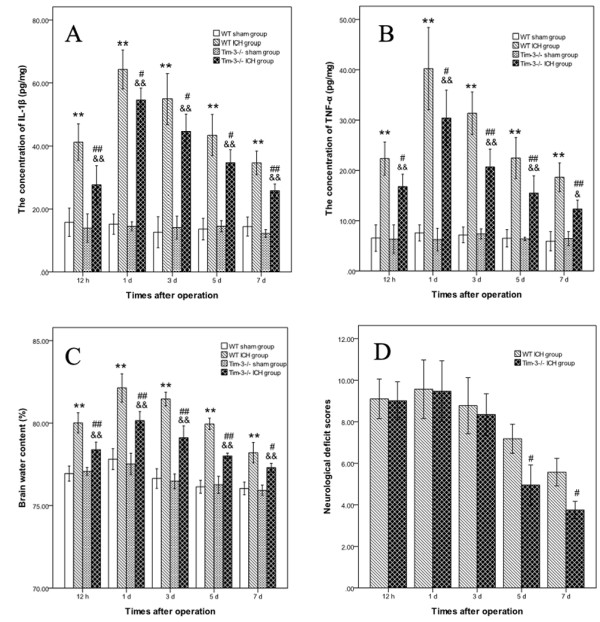
**The change of IL**-**1β**, **TNF**-**a,brain water content and neurological deficit scores (NDS) after intracerebral hemorrhage (ICH). A)** indicates the change of the concentration of IL-1β in the peri-hematomal brain tissues at 12 hours andat 1, 3, 5 and 7 days post-ICH. **B)** shows the change of the concentration of TNF-a. **C)** displays the change of brain water content. **D)** shows the NDS between the Tim-3^−/−^ ICH group and the wild type (WT) ICH group. The WT ICH group (n = 6) versus the WT sham group (n = 6): **P <0.01; The Tim-3^−/−^ ICH group (n = 6) versus the Tim-3^−/−^ sham group (n = 6): &&*P* < 0.01; The Tim-3^−/−^ ICH group (n = 6) versus the WT ICH group (n = 6): ^#^*P* < 0.05, ^##^*P* < 0.01.

IL-1β and TNF-a are two main proinflammatory cytokines of microglial source, which represent the level of brain inflammation [19,20]. We detected the concentration of IL-1β and TNF-a in the peri-hematomal brain tissues at 12 hours and at 1, 3, 5 and 7 days post-ICH. Both of them elevated obviously in the WT ICH group at all tested time points. There was significant difference between the WT ICH group and the WT sham group (*P* < 0.01). IL-1β and TNF-a increased at 12 hours after ICH, and peaked at Day 1, then decreased at Day 3 (Figure 3A,B). We also measured the mice brain water content to judge the degree of brain edema. Brain water content heightened at 12 hours after ICH, peaked at Day 1 and Day 3, and then gradually declined. There was a significant difference between the WT ICH group and the WT sham group (*P* < 0.01) (Figure 3C).

### Positive correlation of expression of Tim-3 with IL-1β, TNF-a and brain water content

**Figure 4:**
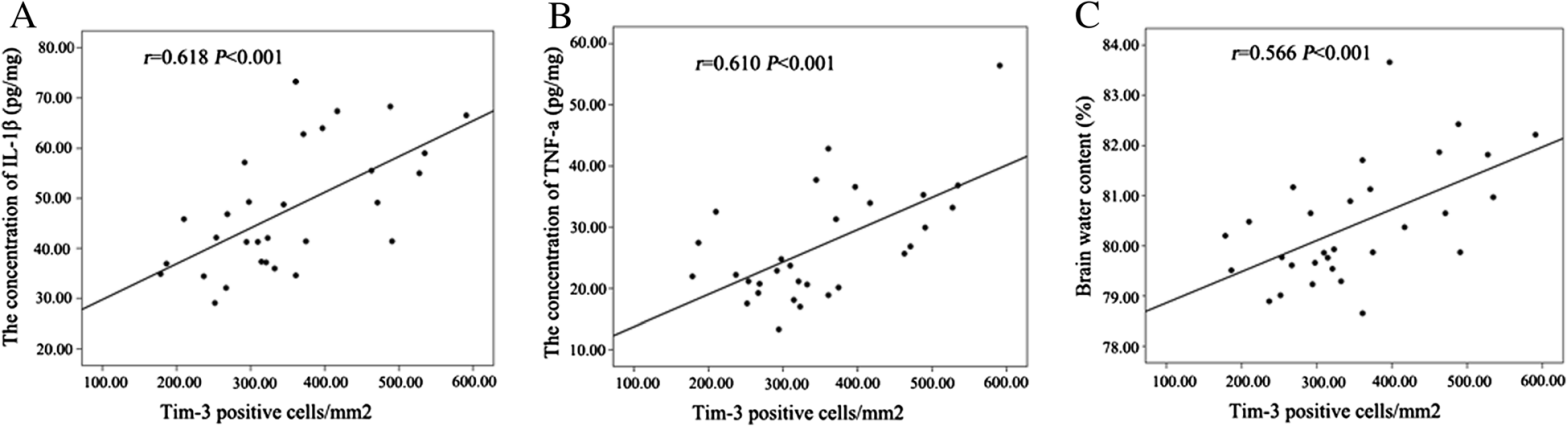
**The correlation between the expression of Tim**-**3 and IL**-**1β**, **TNF**-**a**, **brain water content. A)**, **B)** and **C)** indicate that the expression of Tim-3 was positively correlated with IL-1β (*r* = 0.618, *P <*0.001), TNF-a (*r* = 0.610, *P <*0.001) and brain water content (*r* = 0.566, *P* = 0.001).

We analyzed the correlation between expression of Tim-3 and IL-1β, TNF-a and brain water content. The results show that expression of Tim-3 was positively correlated with IL-1β (*r* = 0.618, *P <*0.001), TNF-a (*r* = 0.610, *P <*0.001) and brain water content (*r* = 0.566, *P* = 0.001)(Figure 4A,B,C).

### The brain inflammatory response, brain edema and neurologic function in the Tim-3^−/−^ ICH mice

To further prove the effects of Tim-3 on brain inflammation, brain edema and neurologic function, we compared the concentration of IL-1β and TNF-a, brain water content and NDS in the WT ICH group with that in the Tim-3^−/−^ ICH group. The concentration of both IL-1β and TNF-a in the peri-hematomal brain tissues notably decreased in the Tim-3^−/−^ ICH group, and there was significant difference compared to that in the WT ICH group (the Tim-3^−/−^ ICH group versus the WT ICH group, IL-1β: 12 h *P <*0.01; 1d *P <*0.05; 3 d *P <*0.05; 5d *P <*0.05; 7d *P <*0.01. TNF-a: 12 h *P <*0.05; 1d *P <*0.05; 3d *P <*0.01; 5d *P <*0.01; 7d *P <*0.01) (Figure 3A,B). Brain water content also markedly declined in the Tim-3^−/−^ ICH group in all tested time points (the Tim-3^−/−^ ICH group versus the WT ICH group, 12 h *P <*0.01; 1d *P <*0.01; 3 d *P <*0.01; 5d *P <*0.01; 7d *P <*0.05.) (Figure 3C). At 12 hours and day 1, there was no difference of NDS between the Tim-3^−/−^ ICH group and the WT ICH group. At 3 days, the NDS of the Tim-3^−/−^ ICH group was lower than that of the WT ICH group, but there was no statistical difference. At 5 and 7 days, there was a significant difference between two groups (*P* < 0.05) (Figure 3D), which meant that blockage of expression of Tim-3 could improve the neurological deficit after ICH.

### The change of astrocytes, neutrophils and microglia/macrophages after intracerebral hemorrhage

**Figure 5:**
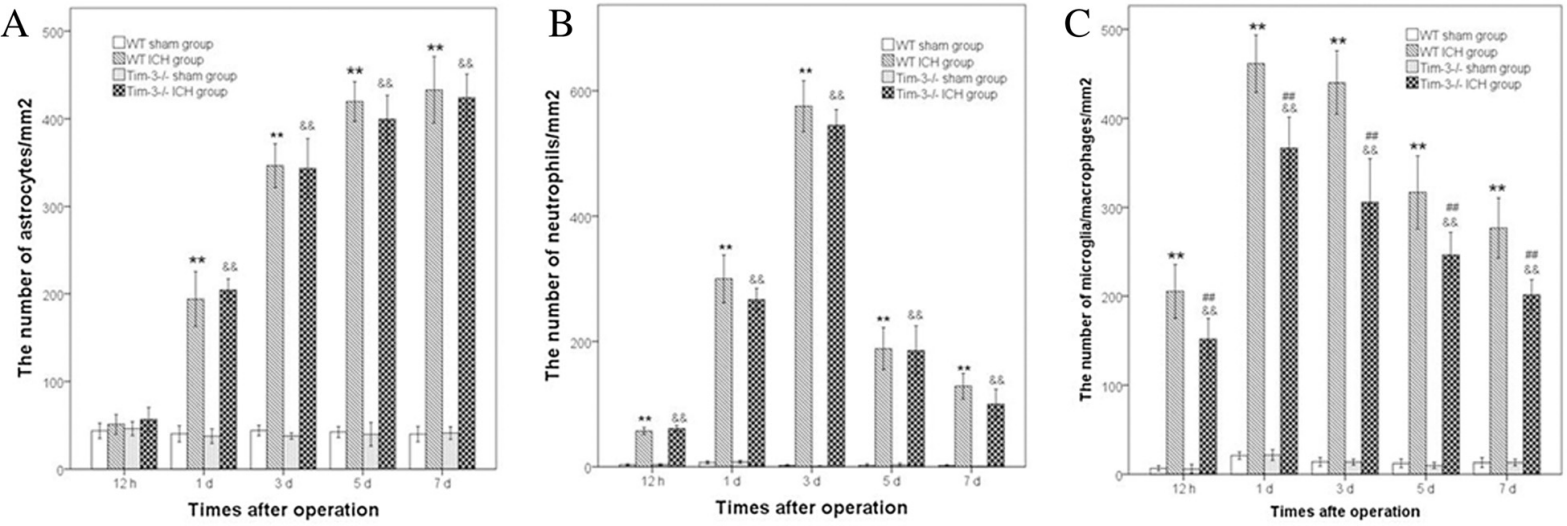
**The change of the number of astrocytes**, **neutrophils and microglia**/**macrophages after intracerebral hemorrhage (ICH).A)** indicates the change in the number of astrocytes in the peri-hematomal brain tissues at 12 hours and at 1, 3, 5 and 7 days post-ICH. **B)** shows the change in the number of neutrophils. **C)** displays the change in the number of microglia/macrophages in the wild type (WT) ICH group (n = 6) versus the WT sham group (n = 6): **P <0.01; The Tim-3^−/−^ ICH group (n = 4) versus the Tim-3^−/−^ sham group (n = 4): &&*P <*0.01; the Tim-3^−/−^ ICH group (n = 4) versus the WT ICH group (n = 6): ^##^*P <*0.01.

We investigated the number of astrocytes, neutrophils and microglia/macrophages in all groups using immunofluorescence staining. The results indicated that the number of astrocytes, neutrophils and microglia/macrophages all increased after ICH. But the peak times of the three cells were different. The number of astrocytes elevated at Day 1, peaked at Day5 and Day 7. The number of neutrophils rose at 12 hours, peaked at Day 3, and then gradually declined. But the number of astrocytes and neutrophils were not different between the WT ICH group and the Tim-3^−/−^ ICH group. The number of microglia/macrophages increased at 12 hours and peaked at Day 1. This variation was the same as that of Tim-3, IL-1β and TNF-a. In Tim-3^−/−^ ICH mice, the number of microglia/macrophages was obviously less than that of WT ICH mice and there was a significant difference between the two groups (*P* < 0.01) (Figure 5).

## Discussion

In the present study we wanted to prove the effect of Tim-3 on brain inflammation after ICH. Three aspects of this research had to be addressed. The first aspect involved the expression of Tim-3 in the peri-hematomal zone after ICH. The second was related to the cellular resource of Tim-3 expression. The third was concerned with the correlation between the expression of Tim-3 and the severity of inflammatory response and brain edema. Furthermore, we examined the change in the release of inflammatory cytokines, brain edema, neurofunctional impairment and inflammatory cells (astrocytes, neutrophils and microglia/macrophages) in Tim-3^−/−^ ICH mice.

### The expression of Tim-3 in the peri-hematomal zone after ICH

Tim-3 is a new immuno-regulation molecule. It has been proved that Tim-3 extensively expresses itself in innate immune cells, including microglia, monocytes, mast cells, dendritic cells and natural killer cells, and plays complex roles in immune regulation and tolerance [11,13,14,21-23]. Tim-3 is involved in several inflammatory diseases, such as experimental autoimmune encephalomyelitis (EAE) [11], non-obese diabetes [24] and Coxsackievirus B3-induced myocarditis [21]. Anderson *et al*. [14] demonstrated that Tim-3 mRNA levels were much higher in inflamed white matter tissue in patients with multiple sclerosis (MS) and rat MS models, and Tim-3 upregulated on CD11b^+^ peripheral monocytes and resident microglia to promote TNF-a secretion. Zhao *et al*. [25] investigated the expression of Tim-3 in the acute phase of ischemic stroke, and indicated that overexpression of Tim-3 both in brain tissues of ischemia-reperfusion mice and in peripheral blood mononuclear cells of patients with ischemic stroke positively correlated with plasma IL-17 and TNF-α. All these researchers suggested that Tim-3 took parts in inflammatory-immunologic reaction. But it is completely unknown whether or not Tim-3 plays roles in ICH. Now, we have experimentally found that Tim-3 increases in the mouse peri-hematomal brain tissues early after autologous blood injection and progressively increasesand accompanies the development of brain inflammation, suggesting that Tim-3 is also involved in the inflammatory response following ICH.

### The cellular resource of Tim-3 expression

Previous studies have revealed that brain inflammation after ICH was characterized by the soakage of neutrophils and macrophages from blood and activation of microglia in brain tissues [8,26]. Among all inflammatory cells, microglia are the first non-neuronal cells to react to brain damage [27]. The shape of activated microglia is the same as the shape of blood-derived macrophages, so it is impossible to discriminate them from infiltrating macrophages. Microglia are activated at 1 hour after ICH, much earlier than neutrophil infiltration, and the latter appears at 4 to 5 h after ICH [9]. Activated microglia/macrophages can release cytotoxic mediators, such as IL-1β and TNF-a, inducing brain inflammatory reaction and secondary damage [10,15,28]. Blockage of microglia activation with tuftsin fragment 1–3 can attenuate neuroinflammation and brain damage after ICH in rats [24,29]. In this experimental study, we found that the expression of Tim-3 following ICH was preponderant in microglia/macrophages, was lower in neutrophils, and was lowest in astrocytes. Although astrocytes, neutrophils and microglia/macrophages all increased following ICH, but only variation of microglia/macrophages was the same as that of Tim-3, IL-1β and TNF-a. These indicate that Tim-3 can inhibit the activation of microglia/macrophages, but not influence the astrocytes and neutrophils. Tim-3 not only expressed in microglia/macrophages, but also could regulate the function of them.

### The relationship between the expression of Tim-3 and the severity of inflammatory response and brain edema

IL-1β and TNF-a are two important proinflammatory cytokines, which can drive the inflammatory process and aggravate inflammation [30,31]. Although IL-1β and TNF-a are released by many cells, including microglia/macrophages, astrocytes and neurons, the major source of these are activated microglia/macrophages [4,19,20]. Many experimental data have revealed that expression of IL-1β and TNF-a increase after ICH, and are associated with brain edema and brain damage. Increased expression of IL-1β and TNF-a can been detected not only in the central nervous system but also in systemic circulation [32-34]. IL-1β and TNF-α can boost inflammatory reaction in the early stage by promoting secretion of other chemotactic factors and adhesion molecules of the vascular endothelium, which can lead to the early infiltration of macrophages and neutrophils to the injury lesion [35,36]. The levels of TNF-α and IL-1β can represent the severity of brain inflammatory to a great degree. In our study, we found that TNF-α and IL-1β increased at 12 hours post-ICH, peaked at Day 1 and decreased at Day 3 after ICH. The expression of Tim-3 is positively correlated to levels of TNF-α, IL-1β and brain water content. Above all, we can deduce that augmented Tim-3 expression may promote inflammation and brain edema in the peri-hematomal tissues after ICH by regulating the function of microglia/macrophage. This result was supported by three points below. First of all, expression of Tim-3 increased early in the perilesional tissue and predominantly expressed itself in microglia/macrophages after ICH. The number of microglia/macrophages was much lower in Tim-3^−/−^ ICH mice. Furthermore, the secretion of TNF-α and IL-1β elevated in the peri-hematomal tissues following ICH, and microglia/macrophages are the major types of cells to secrete TNF-a and IL-1β. In addition, there was a positive correlation between the expression of Tim-3 and the levels of TNF-α, IL-1β and brain water content. Blocking expression of Tim-3 could not only reduce the levels of TNF-α, IL-1β, brain water content and the number of microglia/macrophages, but also improve neurological function, which also elucidated the effect of Tim-3 on brain inflammatory reaction from another aspect. But the signal pathways of Tim-3 that regulate the function of microglia/macrophages need to be further explored.

## Conclusions

Tim-3 plays an important role in the brain inflammation after ICH by regulating the function of microglia/macrophages and may be a potential treatment target.

## Abbreviations

ELISA: Enzyme-linked immunosorbent assay; ICH: Intracerebral hemorrhage; MABP: Mean arterial blood pressure; NDS: Neurological deficit scores; RT-PCR: Reverse transcription polymerase chain reaction.

## Competing interests

The authors declare that they have no competing interests.

## Authors’ contributions

The research was based on the original idea of CJX and YGL. CJX worked on the development of animal model, immunofluorescent staining, behavioral studies, and data analysis and drafted the manuscript. TW worked on the development of animal model, behavioral studies and real-time RT PCR. SC worked on the development of animal model, behavioral studies, ELISA and brain water content. YGL provided the equipment and wrote the manuscript. All authors read and approved the final manuscript.
